# The Mediation Effect of Body Composition on the Association Between Menopause and Hyperuricemia: Evidence From China National Health Survey

**DOI:** 10.3389/fendo.2022.879384

**Published:** 2022-06-10

**Authors:** Huijing He, Li Pan, Feng Liu, Xiaolan Ren, Ze Cui, Lize Pa, Jingbo Zhao, Dingming Wang, Jianwei Du, Hailing Wang, Xianghua Wang, Xia Peng, Chengdong Yu, Ye Wang, Guangliang Shan

**Affiliations:** ^1^ Department of Epidemiology and Statistics, Institute of Basic Medical Sciences, Chinese Academy of Medical Sciences & School of Basic Medicine, Peking Union Medical College, Beijing, China; ^2^ Department of Chronic and Noncommunicable Disease Prevention and Control, Shaanxi Provincial Center for Disease Control and Prevention, Xi’an, China; ^3^ Department of Chronic and Noncommunicable Disease Prevention and Control, Gansu Provincial Center for Disease Control and Prevention, Lanzhou, China; ^4^ Department of Chronic and Noncommunicable Disease Prevention and Control, Hebei Provincial Center for Disease Control and Prevention, Shijiazhuang, China; ^5^ Department of Chronic and Noncommunicable Disease Prevention and Control, Xinjiang Uyghur Autonomous Region Center for Disease Control and Prevention, Urumqi, China; ^6^ Department of Epidemiology and Statistics, School of Public Health, Harbin Medical University, Harbin, China; ^7^ Department of Chronic and Noncommunicable Disease Prevention and Control, Guizhou Provincial Center for Disease Control and Prevention, Guiyang, China; ^8^ Department of Chronic and Noncommunicable Disease Prevention and Control, Hainan Provincial Center for Disease Control and Prevention, Haikou, China; ^9^ Department of Chronic and Noncommunicable Disease Prevention and Control, Inner Mongolia Autonomous Region Center for Disease Control and Prevention, Hohhot, China; ^10^ Integrated Office, Institute of Biomedical Engineering, Chinese Academy of Medical Sciences, Peking Union Medical College, Tianjin, China; ^11^ Department of Chronic and Noncommunicable Disease Prevention and Control, Yunnan Provincial Center for Disease Control and Prevention, Kunming, China; ^12^ School of Population Medicine and Public Health, Chinese Academy of Medical Sciences and Peking Union Medical College, Beijing, China

**Keywords:** mediation analysis, excess adiposity, women health, menopause, serum uric acid

## Abstract

Reproductive factors have been demonstrated to be associated with hyperuricemia. Body composition is an essential determinant influencing serum uric acid (SUA), but it is largely unknown whether increased SUA was influenced by changed body composition during the menopausal transition. As a secondary analysis of China National Health Survey from 2012-to 2017, this study included 18,997 women aged 20 to 80. Menarche age and menopause information were collected by questionnaire interview. Body mass index (BMI), body fat percentage (BFP), fat mass index (FMI), and fat-free mass index (FFMI) were used as body composition indexes. Hyperuricemia was defined as SUA higher than 360μmol/L (approximately 6 mg/dl). Mediation analysis was performed to explore the direct and indirect effects of menopause on hyperuricemia. A 1:2 age-matched case-control data set (n=6202) was designed to control age-related confounders and was used in multivariable analyses. After adjustment of covariates, postmenopausal women had 14.08 (10.89-17.27) μmol/L higher SUA than their premenopausal counterparts. Overweight/obesity and higher levels of BFP, FMI, and FFMI were all found to be positively associated with hyperuricemia. The mediation analysis showed that the total effect of menopause on hyperuricemia was positive, but was substantially mediated by body composition indexes. Forty-five percent of the total effect can be attributed to the indirect effect mediated by BMI (OR for the natural indirect effect (NIE): 1.09, 95%CI: 1.04-1.13), and over 80% mediated by BFP (OR for NIE: 1.23, 95%CI: 1.16-1.29). However, FFMI did not present the mediated role in the association (OR for NIE: 0.99, 95%CI: 0.96-1.02). The findings revealed that body composition, especially the fat mass indexes, significantly mediated the association between menopause and hyperuricemia. The role of body composition as mediator constitutes clinical and public health significance that should be recognized and considered in healthcare for women experiencing their menopause transition.

## Introduction

Serum uric acid (SUA) is the catabolic product of exogenous dietetic compounds and endogenous purines (1). High-purine diets or other dietary factors that induce the degradation of purine nucleotides may increase serum urate (2–4). About 70% of daily SUA excretion occurs via the kidney, and in 5%-25% of humans, impaired renal excretion leads to hyperuricemia (5). Our previous work revealed an around 20% hyperuricemia prevalence among adults in mainland China (6). Hyperuricemia is a necessary precursor to develop gout and is also associated with an increased risk of cardiometabolic morbidities and premature mortality (7, 8). Although hyperuricemia is much more common in men than in women (6), the incidence of hyperuricemia increases in postmenopausal women, whereas premenopausal females are possibly protected by the uricosuric effect of estrogen (9).

In previous epidemiological studies, reproductive factors, such as earlier age at menarche or menopause transition (MT) were found to be associated with elevated SUA levels and gout (10, 11). However, there were conflicting conclusions. For example, using data from the US National Health and Nutrition Examination Survey (NHANES) from1988 to 1994, menopause was found to be associated with higher SUA levels (12), but another study using data from NHANES 1999 to 2010 concluded that the increased prevalence of hyperuricemia was not due to menopause but rather to aging (13).

The relationship between adiposity and SUA has been demonstrated by substantial evidence (14–16). Our previous analysis also indicated that adiposity is related to SUA and hyperuricemia (6, 17). Given that body mass index (BMI) is not able to distinguish adiposity between fat-free mass (FFM) and fat mass (FM), the current study uses BMI, fat mass index (FMI), and fat-free mass index (FFMI) as the measurement of body composition. The three indexes are all standardized by stature, and the contribution of both FM and FFM to BMI can be easily assessed (18, 19).

As body mass distribution may change during MT period (20), there could be both direct and indirect effects of menopause on SUA levels: body composition may act as a mediator on the pathway from menopause to hyperuricemia. The importance of mediation analysis relies on the need to disentangle the different pathways that explain the effect of exposure on the outcome (21). As adiposity is one of the modifiable risk factors for hyperuricemia, it is of interest to know what the effect of menopause would be if weight loss occurred. However, whether body composition mediated the effect of reproductive factors on hyperuricemia is still unclear.

Here, using data from a nationally representative survey, the China National Health Survey (CNHS), we for the first time performed mediation analysis to test the mediated effect of diverse body composition indexes on the association between menopause and hyperuricemia.

## Material and Methods

### Study Design and Population

Data of CNHS were used for this study. CNHS is a national representative health survey conducted from 2012 to 2017 among general Chinese population. Using a multi-stage, stratified sampling method, participants aged 20 to 80, living locally for at least one year, were recruited in the survey. Pregnant or lactating women, soldiers on active service, and patients with severe mental or physical conditions were excluded. Standardized questionnaire interview, physical examination, and biochemical tests were conducted. More details for CNHS were available in our previous publication (22). The study has been carried out in accordance with the Declaration of Helsinki. Ethical approval was obtained from the Bioethical Committee of Institute of Basic Medical Sciences, Chinese Academy of Medical Sciences (No.029-2013). All participants provided written informed consent before the survey.

### Outcome Measurement and Definitions

Venous blood samples were drawn after an overnight fast. Separated plasma or serum was frozen in aliquots and stored at -80°C until thawed for the first time for the analyses. SUA was measured by oxidization with the specific enzyme uricase on a Chemistry Analyzer (ROCHE Cobas8000C701, USA).

Hyperuricemia was defined as SUA higher than 360μmol/L (approximately 6 mg/dl) in women, consistent with other previous studies (23).

### Reproductive Factors and Body Composition Measurement

Reproductive information included age of menarche and menopausal status; both were self-reported in the face-to-face questionnaire interview. Participants included in the survey were asked whether they had at least one menstrual bleeding in the past year to define menopause, excluding those caused by medications, hormones, medical conditions, or surgical procedures.

During the anthropometric measurements, participants wore light clothing and were barefoot. Height was measured to the nearest 0.1 cm using a fixed stadiometer. Weight, body fat percentage (BFP), FM, and FFM were measured by a body composition analyzer (TANITA BC-420, Japan), with the accuracy on a decimal level. Body composition indexes in this study included BMI, BFP, FMI, and FFMI. BMI was calculated as weight divided by height squared (kg/m^2^). FMI was calculated as FM in kilograms divided by height in square meters (kg/m^2^), and FFMI was fat-free mass in kilograms divided by height in square meters (kg/m^2^). Underweight was defined as BMI < 18.5 kg/m^2^, normal weight was BMI between 18.5 kg/m^2^ and < 24 kg/m^2^, overweight was BMI ≥ 24 kg/m^2^ but < 28 kg/m^2^, and obesity was BMI ≥ 28 kg/m^2^, according to the recommendation suitable for Chinese population (24).

### Assessment and Definition of Covariates

The demographic information (sex, age, educational levels, personal annual income) and health-related lifestyle factors (cigarette smoking, alcohol consumption, physical activity) were collected by trained investigators using a standardized questionnaire (22). The definition of cigarette smoking, alcohol drinking, and physical activity was descripted previously (22).

### Methodology of Mediation Analysis

Given the association between body composition, menopause,and hyperuricemia, mediation analyses were conducted to examine whether body composition mediated the association between menopause (the independent variable) and hyperuricemia (the outcome variable).

For demonstration purposes, let the mediator, exposure, and outcome all have binary values. The total effect (TE) for a subject is defined as the difference between the counterfactual outcomes at the exposed and unexposed levels. The natural direct effect (NDE) for a subject is defined as the difference between the counterfactual outcomes at the two exposed levels when sets the mediator to the natural level when there is no exposure. The natural indirect effect (NIE) is defined as the difference between the counterfactual outcomes at the two mediators’ levels when sets the exposure to positive value. These definitions lead to the following conventional two-way decomposition of the TE: TE=NDE+NIE. The percentage of total effect that is mediated (PM) is computed as NIE/TE×100%.

### Statistical Analyses

Our analyses restricted to female adults with no missing values on reproductive, body composition, and SUA information. The flow chart of sample selection is available in **Supplementary Figure S1**. The final analytic sample included 18,997 women.

**Figure 1:**
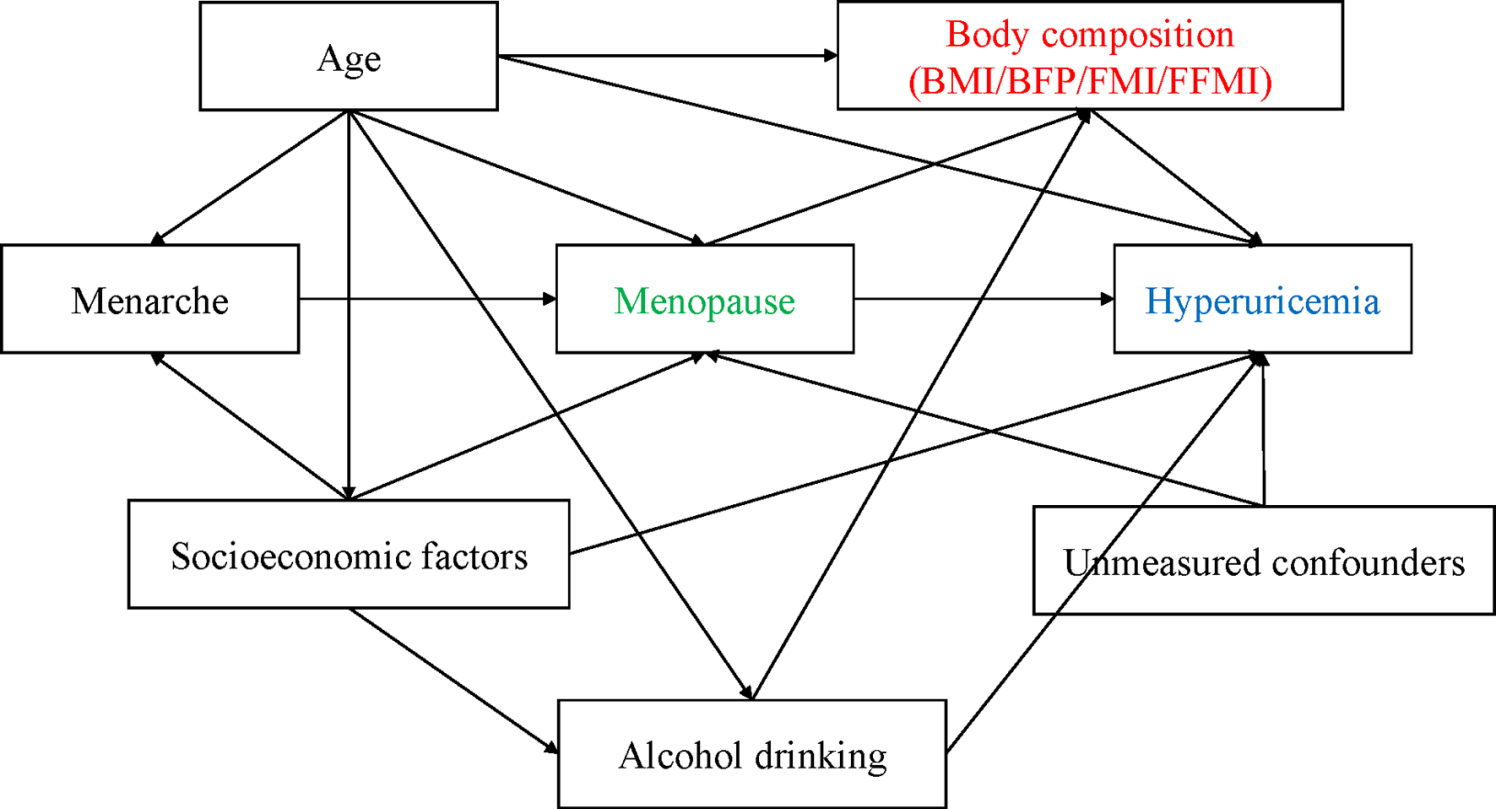
The relationships between covariates and hyperuricemia demonstrating by directed acyclic graph (DAG).

Continuous variables were presented using median and interquartile range (IQR), and frequency with percentage were used for categorical variables. A directed acyclic graph (DAG) was drawn to help establish the analysis strategy (**Figure 1**). Restrict cubic spline (RCS) models were used to examine the dose-response relationship between age, body composition, and hyperuricemia risk. Given similar results yielded by the RCS models among adiposity indexes, we only presented the dose-response relationship between BMI and the risk of hyperuricemia. As age is an essential unbalanced confounder or modifier between premenopausal and postmenopausal women, we performed the analyses based on two data sets: one is the unmatched primary data using all sample’s information, another is a 1:2 age-matched case-control data set containing 2070 cases and 4132 controls. The basic characteristics of the case-control data set are shown in **Supplementary Table S1**. We fitted general linear regression models (GLMs) in which SUA was the dependent outcome variable, reproductive risk factors, and body composition indexes were independent variables with adjustment of potential confounders. Multivariable logistic regression models (conditional logistic model for matched case-control design) were fitted to examine the association between independent risk factors and the outcome (hyperuricemia). We further did stratification analysis to examine whether the estimated regression coefficients would be modified under conditional health status. It helps us demonstrate the necessity of performing mediation analysis, which was performed based on the case-control data set because of its better performance on confounding control. The grouping criteria of BFP (32%), FMI (7 kg/m^2^), and FFMI (15 kg/m^2^) were based on their median values and the areas under the receiver operation characteristic curves (AUCs) in predicting hyperuricemia.

Trying to avoid causal inversion caused by body composition change after MT, we performed sensitivity analysis restricting on women who had experienced their MT within 5 years, comparing with their premenopausal counterparts.

For all coefficients and odds ratio (OR) estimates, we calculated their 95% confidence intervals (CIs). All statistical analyses were performed using SAS version 9.4 (SAS institute, Cary, NC).

## Results

### General Characteristics

A total of 18,997 women (mean age 48.30±13.17 years, ranged from 20 to 80 years) were included in the final analytic sample. Of them, 2070 (10.90%) participants had hyperuricemia. The general characteristics are shown in **Table 1**. The average levels of SUA in the overall population were 277.87±66.92 μmol/L, 406.91±46.75μmol/L in the hyperuricemia group and 262.09±49.74μmol/L in the non- hyperuricemia group. Hyperuricemia patients were older, had higher BMI, FM, FFM, FMI, and FFMI than their non-hyperuricemia counterparts (all P values < 0.001), and with higher proportion of postmenopausal status (P<0.001). The basic characteristics of the 1:2 age-matched data set are shown in **Supplementary Table S1**.

**Table 1:** Basic characteristics of the study population in CNHS (n=18997).

Characteristics	HUA (n=2070)		Non-HUA (n=16927)		P
**Demographic information**					
Age, year (median, IQR)	54.40	17.40	47.90	18.90	<0.001
*Age-group (n, %)*					<0.001
20-	159	7.77	1888	92.23	
30-	201	6.43	2927	93.57	
40-	379	7.42	4730	92.58	
50-	636	13.42	4102	86.58	
60-80	695	17.48	3280	82.52	
*Marital status (n, %)*					<0.001
Unmarried	85	7.96	983	92.04	
Inmarriage	1763	10.73	14667	89.27	
Others	220	14.97	1250	85.03	
*Residential areas (n, %)*					<0.001
Urban	1489	12.16	10760	87.84	
Rural	578	8.61	6132	91.39	
*Education (n, %)*					<0.001
Illiterate/Elementary school	714	12.45	5020	87.55	
High school	942	10.83	7755	89.17	
College or above	410	9.08	4107	90.92	
*Annual personal income (CHY) (n, %)*					0.460
<10000	481	10.77	3984	89.23	
10000-	934	11.33	7312	88.67	
30000-	465	10.45	3986	89.55	
≥ 50000	147	11.23	1162	88.77	
**Health related life-style factors**					
*Physical activity (n, %)*					0.001
Low	446	10.04	3997	89.96	
Moderate	1475	11.44	11418	88.56	
Heavy	149	8.97	1512	91.03	
*Ever smoke (n, %)*	201	9.39	1939	90.61	0.017
Never smoke (n, %)	1869	11.11	14961	88.89	
*Ever-alcohol drink (n, %)*	493	10.21	4334	89.79	0.071
Never drink (n, %)	1576	11.15	12554	88.85	
**Body composition indexes**					
BMI (kg/m^2^) (median, IQR)	25.63	4.78	23.08	4.51	<0.001
*BMI category (n, %)*					<0.001
Under/normal weight	670	6.09	10325	93.91	
Overweight	868	14.81	4991	85.19	
Obesity	532	24.83	1611	75.17	
BFP (%) (median, IQR)	36.30	7.00	31.80	7.90	<0.001
Fat free mass (kg) (median, IQR)	37.50	4.60	36.40	4.50	<0.001
Fat mass (kg) (median, IQR)	22.60	8.70	18.00	7.90	<0.001
FMI (kg/m^2^) (median, IQR)	9.33	3.55	7.37	3.25	<0.001
FFMI (kg/m^2^) (median, IQR)	15.33	1.15	14.85	1.24	<0.001
**Reproductive factors**					
*Menarche age (median, IQR)*	15	3	15	3	0.146
≤12	222	11.14	1771	88.86	0.708
>12	1829	10.86	15008	89.14	
*Postmenopause (n, %)*	1297	15.44	7101	84.56	<0.001
Premenopause (n, %)	773	7.29	9826	92.71	
**Clinical characteristics**					
eGFR (ml/min/1.73m^2^) (median, IQR)	87.70	25.62	101.55	27.41	<0.001
Creatinine (μmol/L) (median, IQR)	68.50	14.90	62.00	12.50	<0.001

CNHS, China National Health Survey; HUA, hyperuricemia; IQR, Interquartile range; BMI, body mass index (kg/m^2^); BFP, body fat percentage (%); FMI, fat mass index (kg/m^2^); FFMI, fat free mass index (kg/m^2^); eGFR, estimated glomerular filtration rate.

**Figure 2:**
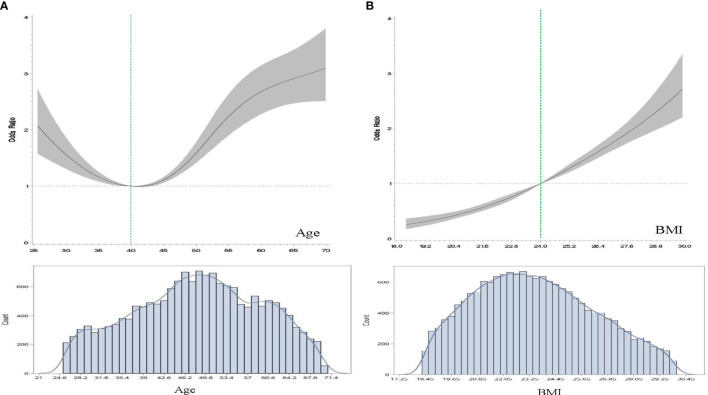
The dose-response relationships of age, BMI with the odds ratios (OR) of hyperuricemia using restricted cubic spline (RCS) modeling. **(A)**, The association between age and hyperuricemia. The model was adjusted for body mass index (BMI, kg/m^2^), residential areas, educational levels, study sites and alcohol drinking status. **(B)**, The association between BMI and hyperuricemia. The model was adjusted for age, residential areas, educational levels, study sites, and alcohol drinking status.

As shown in **Figure 2A**, there was a U-shape relationship between age and the OR of hyperuricemia. Stratified by age at 40, women younger or older both had increased risk of hyperuricemia.

### Associations of Body Composition, Reproductive Factors With SUA and Hyperuricemia

**Table 2** shows the associations of adiposity, reproductive factors with SUA levels in both unmatched and age-matched case-control analyses. After the adjustment of covariates, the average SUA levels showed a 22.09 μmol/L increase in postmenopausal women in the unmatched analysis, whereas this number is down to 14.08 in the case-control analysis. Early menarche was found to be slightly associated with elevated SUA, the regression coefficient in the unmatched analysis was also greater than the case-control analysis (-0.96, -1.36 to -0.55 vs. -0.76, -1.50 to -0.01). Adiposity indexes, such as BMI, BFP, FMI, and FFMI all presented positive associations with SUA. FFMI seemed to have greater effect than the other adiposity indexes, with 14.2 μmol/L higher SUA as per unit change of FFMI. The stratification analysis by adiposity suggested that the association between reproductive factors and SUA seemed to be stronger in the higher adiposity group (**Table 2**. Case-control analysis).

**Table 2:** The effect of reproductive and body composition factors on serum uric acid among female participants in CNHS.

	Un-matched analysis				1:2 Age-matched analysis			
	B	95%CI		P	B	95%CI		P
**Overall**								
Menopause	22.09	19.37	24.81	<0.001	14.08	10.89	17.27	<0.001
Menarche age	-0.96	-1.36	-0.55	<0.001	-0.76	-1.50	-0.01	0.047
BMI	5.51	5.17	5.85	<0.001	4.17	3.75	4.59	<0.001
BFP	2.87	2.03	3.70	<0.001	2.94	2.65	3.22	<0.001
FMI	7.17	5.85	8.49	<0.001	5.76	5.19	6.34	<0.001
FFMI	17.03	16.11	17.96	<0.001	14.19	12.51	15.88	<0.001
**BMI<24**								
Menopause	20.98	17.48	24.48	<0.001	7.48	1.60	13.37	0.013
Menarche age <12	-0.59	-1.19	0.01	0.054	-1.17	-2.47	0.13	0.077
**BMI≥24**								
Menopause	22.22	17.62	26.81	<0.001	11.39	5.88	16.90	<0.001
Menarche age <12	-0.68	-1.43	0.07	0.075	-1.58	-2.82	-0.34	0.013
**BFP<32**								
Menopause	24.43	20.65	28.20	<0.001	7.66	1.09	14.23	0.022
Menarche age <12	-0.44	-1.36	0.48	0.351	-2.82	-4.23	-1.41	<0.001
**BFP≥32**								
Menopause	21.05	16.81	25.30	<0.001	17.48	11.88	23.08	<0.001
Menarche age <12	-0.38	-1.89	1.12	0.619	-1.37	-2.56	-0.17	0.025
**FMI<7**								
Menopause	5.06	-12.10	22.22	0.563	-41.98	-115.54	31.58	0.263
Menarche age <12	-1.09	-1.64	-0.54	<0.001	1.91	6.64	0.79	0.430
**FMI≥7**								
Menopause	20.88	17.55	24.22	<0.001	17.46	12.88	22.03	<0.001
Menarche age <12	-0.73	-1.79	0.33	0.179	-1.24	-2.24	-0.23	0.016
**FFMI<15**								
Menopause	17.15	10.61	23.68	<0.001	-9.45	-35.54	16.64	0.478
Menarche age <12	0.10	-0.98	1.18	0.860	0.25	-2.77	3.28	0.870
**FFMI≥15**								
Menopause	-3.38	-28.49	21.73	0.792	21.70	16.14	27.26	<0.001
Menarche age <12	2.67	-1.09	6.43	0.164	-1.49	-2.83	-0.16	0.029

The unmatched model was adjusted by age, educational levels, residential areas, study sites, and income. The age-matched analysis was adjusted by the same covariates except for age. CNHS, China National Health Survey; B, regression coefficient. CI, confidence interval. BMI, body mass index (kg/m^2^); BFP, body fat percentage (%); FMI, fat mass index (kg/m^2^); FFMI, fat free mass index (kg/m^2^).

There was a dose-response linear relationship between BMI and the OR of hyperuricemia (**Figure 2B**). Other body composition indexes had similar results. Results from the logistic regression models revealed that adiposity and menopause were positively associated with increased risk of hyperuricemia (**Table 3**). The obese female had a 4.43-fold higher risk of hyperuricemia than their normal weight counterparts. Postmenopausal women had a 55% (22%-98%) higher risk of hyperuricemia than premenopausal females. After stratified by adiposity status, the association between menopause and hyperuricemia were positive in the higher adiposity group (BMI≥24, BFP≥32, FMI≥7, and FFMI≥15).

**Table 3:** The results of logistic regression modeling examine the effect of reproductive and body composition factors on hyperuricemia.

	Un-matched analysis (n=18997)				1:2 Age-matched analysis (n=6202)			
	OR	95%CI		P	OR	95%CI		P
**Overall**								
Overweight	2.548	2.247	2.889	<0.001	2.658	2.307	3.063	<0.001
Obesity	4.941	4.200	5.812	<0.001	5.432	4.519	6.529	<0.001
Higher BFP	3.254	2.681	3.950	<0.001	3.405	2.890	4.013	<0.001
Higher FMI	3.405	2.830	4.098	<0.001	3.664	3.134	4.282	<0.001
Higher FFMI	2.571	2.323	2.845	<0.001	2.673	2.350	3.042	<0.001
Menopause	1.884	1.614	2.198	<0.001	1.554	1.222	1.975	<0.001
Menarche age <12	1.171	1.016	1.349	0.031	1.231	0.995	1.524	0.056
**BMI<24**								
Menopause	1.999	1.538	2.598	<0.001	1.093	0.642	1.861	0.742
Menarche age <12	1.241	0.981	1.571	0.071	1.209	0.803	1.823	0.364
**BMI≥24**								
Menopause	1.845	1.554	2.190	<0.001	1.430	1.007	2.029	0.046
Menarche age <12	1.151	0.992	1.336	0.063	1.093	0.774	1.544	0.613
**BFP<32**								
Menopause	1.989	1.542	2.565	<0.001	1.629	0.927	2.861	0.090
Menarche age <12	1.342	1.007	1.787	0.045	1.231	0.840	1.803	0.286
**BFP≥32**								
Menopause	1.783	1.504	2.115	<0.001	1.515	1.058	2.169	0.023
Menarche age <12	1.142	0.944	1.380	0.166	0.937	0.656	1.339	0.721
**FMI<7**								
Menopause	2.288	1.531	3.419	<0.001	1.314	0.447	3.867	0.620
Menarche age <12	1.178	0.827	1.679	0.356	1.006	0.572	1.771	0.983
**FMI≥7**								
Menopause	1.805	1.544	2.110	<0.001	1.457	1.093	1.944	0.010
Menarche age <12	1.204	1.021	1.421	0.029	1.123	0.856	1.472	0.402
**FFMI<15**								
Menopause	1.969	1.457	2.661	<0.001	1.116	0.662	1.884	0.680
Menarche age <12	1.288	1.008	1.645	0.044	1.415	0.946	2.117	0.091
**FFMI≥15**								
Menopause	1.931	1.636	2.279	<0.001	1.425	0.999	2.030	0.050
Menarche age <12	1.145	0.973	1.347	0.101	1.057	0.753	1.484	0.750

The conditional logistic regression modeling method was used in the age-matched case-control analysis. The confounders adjusted in the logistic models were age (only in the unmatched analysis), residential areas, study sites, educational levels, and income. OR, odds ratio; CI, confidence interval; BFP, body fat percentage, %; FMI, fat mass index, kg/m^2^; FFMI, fat free mass index, kg/m^2^; BMI, body mass index, kg/m^2^.

### Mediation Effect of Body Composition

**Figure 3:**
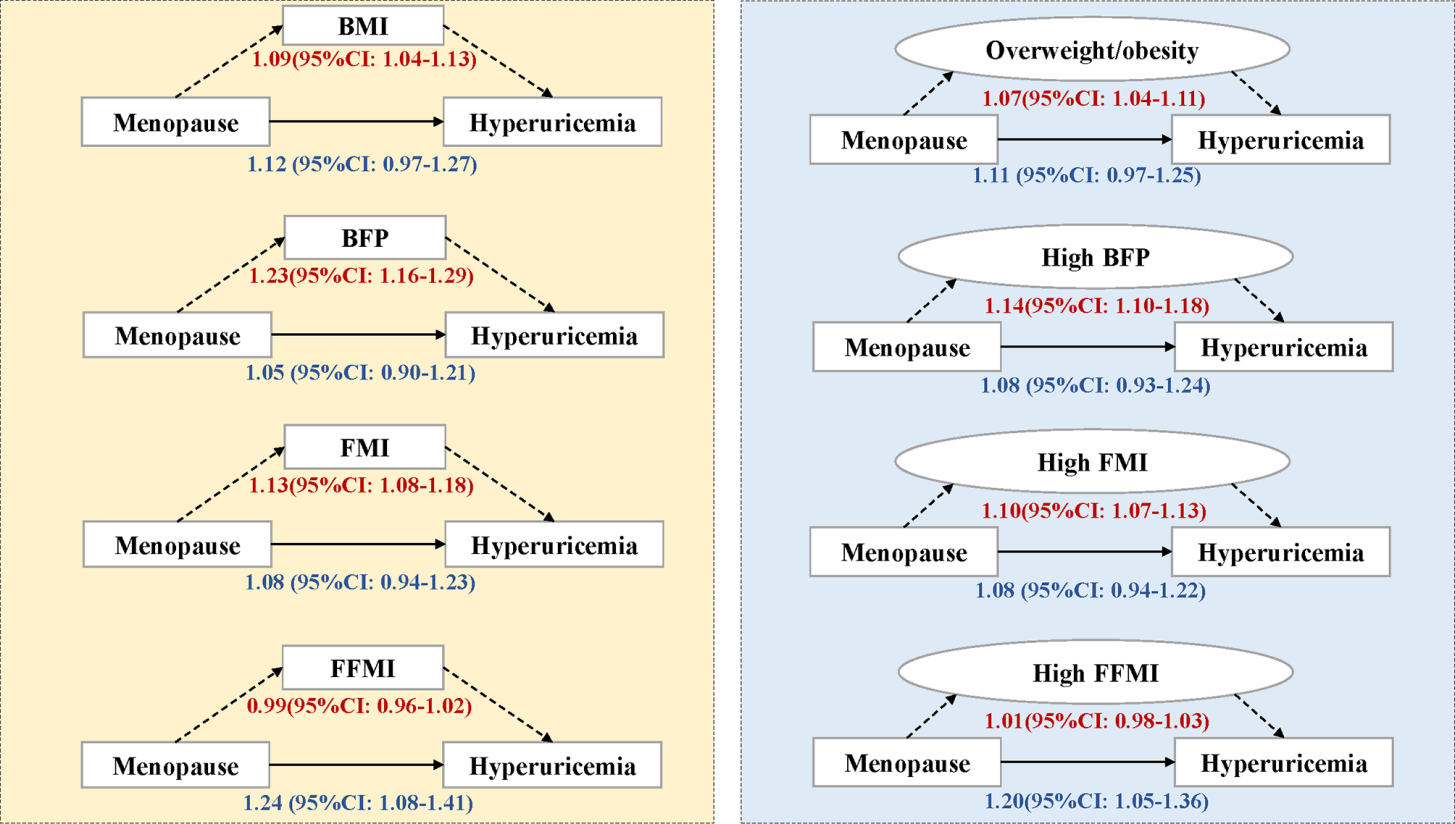
Mediation analysis of body composition on the association between menopause and hyperuricemia. The number represents odds ratios and their 95% confidence intervals in the direct or indirect effect estimates. BMI, body mass index, kg/m^2^; BFP, body fat percentage, %; FMI, fat mass index, kg/m^2^; FFMI, fat free mass index, kg/m^2^; CI, confidence interval. Overweight/obesity refers to BMI≥24, high BFP refers to BFP>32%, high FMI refers to FMI>7, and high FFMI refers to FFMI>15.

The results of mediation analyses are presented in **Figure 3** and **Supplementary Table S2**. The total effect of menopause on hyperuricemia estimated by OR was 1.22 (95%CI: 1.05-1.39) through the BMI mediated pathway. However, when we decomposed the effect into NDE and NIE, the effect was mainly mediated by BMI or overweight/obesity, which was about 45% (15.62%-75.02%) of the effect is mediated through BMI. Likewise, the effect of menopause through other body composition indexes showed similar results except for FFMI, of which the NDE was positive (OR: 1.24, 95%CI: 1.08-1.41). The mediation effect of BFP was the largest among the adiposity indexes where 81.7% of the effect can be explained by the indirect pathway. The results of sensitivity analyses are shown in **Supplementary Table S2**, which were consistent with the primary analyses.

## Discussion

In this large sample of Chinese female adults in CNHS, we found that increased body composition and menopausal status are associated with elevated SUA. Body composition indexes, especially those indicating fat mass distribution, significantly mediated the association between menopause and hyperuricemia.

In line with previous studies, we found that the total effect of menopause was positive on influencing hyperuricemia. Higher SUA levels have been observed in postmenopausal women in both European and Asian populations (10, 25). Other reproductive factors, such as early menarche age, was also the study interest in some studies. For example, Bubach et al. suggested that early menarche age may lead to higher prevalence of metabolic cardiovascular risk factors, but this effect was partially mediated by body composition (26). The mechanism behind the influence of early menarche on metabolic health profile may be attributable to endogenous estrogen. Our study also revealed that menarche age was negatively associated with SUA levels; the earlier the menarche age was, the higher SUA levels would be, and this effect was greater among women with higher body composition levels.

The different effect size of menopause on hyperuricemia in the stratification analysis constitutes the need of mediation analyses. First and the foremost, we demonstrated the rationality of the analysis, which relies on the causal role or temporality of several pathways shown in the DAG (**Figure 1**). The causal role of adiposity on hyperuricemia has been well established by previous epidemiological studies (27, 28). The essential question is how to understand the relationship between adiposity and menopause. Investigated by Greendale et al., trying to examine the relationship between MT and body composition change, they investigated women in the SWAN cohort and found that at the start of MT, the rate of fat gain doubled, and lean mass declined (20). The hormonal changes during MT are associated with changes in energy expenditure and energy intake that promote a positive energy balance, resulting in weight gain (29–32). In this study, the main concern is the time sequence of weight change and MT. By secondary design of the age-matched case-control data set, we, to some extent, balanced the age-related confounding between hyperuricemia and non-hyperuricemia group, including potential weight change or reproductive characteristics in different age stage. As a previous study argued that the effect of menopause on increasing SUA was more likely caused by aging, but not menopause itself (13), the age-matched analysis could yield a more reliable conclusion not biased by age. Furthermore, we performed sensitivity analyses restricted on women who experienced their MT within 5 years and observed consistent findings with the primary analyses. Notably, the unmatched analyses tended to yield overestimates of the health effect even with adjustment of age in the multivariable model. Stratified or matching methodology therefore is needed to cope with residual confounding in this scenario.

Illustrated by DAG for the current study, menarche age may be more directly related with menopausal transition, but not elevated hyperuricemia, or through influencing MT to have an indirect effect of hyperuricemia. Under this hypothesis, we did not include menarche age as a covariate in the mediation analyses based on Rothman’s recommendation on confounder’s adjustment (33).

Our study indicated that the effect of menopause on hyperuricemia is mainly attributable to the indirect effect through the change of body fat mass. Epidemiological studies have reported that SUA is correlated with estrogen change in the menstrual cycle and estrogen therapy may lead to a decrease in SUA (5, 12, 34), strengthened the evidence that estrogen change during MT could be the reason of increased risk of hyperuricemia. However, most studies did not distinguish body fat mass and fat free mass, moreover, the multivariable adjustment is not sufficient to eliminate the indirect effect of body composition on the study outcome. Interestingly, there was a difference in the mediation effect among adiposity indexes. Indexes reflecting the fat mass distribution were more likely to influence the association between menopause and hyperuricemia. Supported by previous evidence, MT may accompany the increase of fat mass but decrease of lean mass (20). This finding suggested that more attention should be paid on fat mass control when initiating body weight management in the MT period, rather than simple weight loss.

Postmenopausal life is characterized by increased disturbances from which women are relatively protected during fertile life (35). Menopause has now been suggested as a female-specific cardiovascular risk factor by the American Heart Association (36). The mediation analyses enable assessing to what extent the effect of menopause is explained by the mediator (body composition in this study). It may provide new perspectives for healthcare providers to manage hyperuricemia for women at MT period. Although hyperuricemia is the basis for developing gout, most people with hyperuricemia are asymptomatic and do not develop gout (2). Therefore, initiating disease prevention in the asymptomatic stage may lower the risk of developing gout and other severe conditions and thus reach better health benefits.

The strength of this study is first on its large representative sample. Using multi-stage stratified sampling method, we recruited a community-based general population in mainland China. The rigid design and the use of standardized measurement all through the survey place it particularly well for the health effect estimates and the results from different study sites were comparable. Second, mediation analysis was used to discompose the effect of risk factors on outcome, thus help health professionals to initiate targeted intervention. Third, we used diverse body composition indexes, including BMI, BFP, FMI, and FFMI, representing both fat mass and fat-free mass distribution, to reach more comprehensive health estimates.

The limitations of this study should also be acknowledged. As the original cross-sectional or case-control design, it is difficult to establish the temporal sequences between exposures and outcomes (temporality). Although we have demonstrated the causal relationship between menopause, body composition, and elevated SUA levels, the temporal sequence of adiposity change, and menopause cannot be established. Nevertheless, we performed a matched case-control design based on the original data, trying to balance predominant confounders, so the results are supposed to be more reliable than those directly yielded by the unmatched sample. We also performed the sensitivity analysis and found no reversed conclusion. Another limitation is the unmeasured confounding. As illustrated by DAG, there still are unmeasured confounders in the causal relationships between independent and dependent variables. For example, dietary assessment and hormone replacement therapy experience information were not collected, which may influence SUA levels during MT.

In summary, in addition to supporting a previously reported association between menopause and elevated SUA, our study for the first time provides evidence in a representative large female population that this effect is significantly mediated by body composition. Menopause indirectly affects the risk of hyperuricemia through elevating body adiposity. Furthermore, fat mass, but not fat-free mass, plays a predominant role in this mediation effect.

## Data Availability Statement

The original contributions presented in the study are included in the article/**Supplementary Material**, further inquiries can be directed to the corresponding author.

## Ethics Statement

The studies involving human participants were reviewed and approved by Bioethical Committee of Institute of Basic Medical Sciences, Chinese Academy of Medical Sciences. The patients/participants provided their written informed consent to participate in this study.

## Author Contributions

Conceptualization, HH and GS; methodology, HH; software, HH; validation, HH, GS, and LPan; formal analysis, HH; investigation, LPan, XR, DW, JD, ZC, HW, XW, FL, LPa, XP, YW, CY, GS, and HH; resources, GS, HH, XR, DW, JD, ZC, HW, XW, FL, LPa, XP, YW, and CY; data curation, HH and GS; writing—original draft preparation, HH; writing-review and editing, GS; visualization, HH, YW, and CY; supervision, GS and LPan; project administration, GS, LPan, and HH; funding acquisition, GS and HH. All authors read and approved the final manuscript.

## Funding

This work was funded by the National Key R&D Program of China (Grant No. 2016YFC0900600/2016YFC0900601), the Key Basic Research Program of the Ministry of Science and Technology of China (Grant No. 2013FY114100), the National Natural Science Foundation of China (Grant No.82003531).

## Conflict of Interest

The authors declare that the research was conducted in the absence of any commercial or financial relationships that could be construed as a potential conflict of interest.

## Publisher’s Note

All claims expressed in this article are solely those of the authors and do not necessarily represent those of their affiliated organizations, or those of the publisher, the editors and the reviewers. Any product that may be evaluated in this article, or claim that may be made by its manufacturer, is not guaranteed or endorsed by the publisher.

## References

[1] Fathallah-Shaykh SA Cramer MT. Uric Acid and the Kidney. Pediatr Nephrol (2014) 29(6):999–1008. doi: 10.1007/s00467-013-2549-x 23824181

[2] Dalbeth N Gosling AL Gaffo A Abhishek A. Gout. Lancet (2021) 397(10287):1843–55. doi: 10.1016/S0140-6736(21)00569-9 33798500

[3] Choi HK Curhan G. Soft Drinks, Fructose Consumption, and the Risk of Gout in Men: Prospective Cohort Study. BMJ (2008) 336(7639):309–12. doi: 10.1136/bmj.39449.819271.BE PMC2234536 18244959

[4] Choi HK Atkinson K Karlson EW Willett W Curhan G. Purine-Rich Foods, Dairy and Protein Intake, and the Risk of Gout in Men. N Engl J Med (2004) 350(11):1093–103. doi: 10.1056/NEJMoa035700 15014182

[5] Mumford SL Dasharathy SS Pollack AZ Perkins NJ Mattison DR Cole SR. Serum Uric Acid in Relation to Endogenous Reproductive Hormones During the Menstrual Cycle: Findings From the BioCycle Study. Hum Reprod (2013) 28(7):1853–62. doi: 10.1093/humrep/det085 PMC3685334 23562957

[6] He H Pan L Ren X Wang D Du J Cui Z. The Effect of Body Weight and Alcohol Consumption on Hyperuricemia and Their Population Attributable Fractions: A National Health Survey in China. Obes Facts (2022) 15(2):216–27. doi: 10.1159/000521163 PMC9021635 34839297

[7] Zhu Y Pandya BJ Choi HK. Comorbidities of Gout and Hyperuricemia in the US General Population: NHANES 2007-2008. Am J Med (2012) 125(7):679–87.e1. doi: 10.1016/j.amjmed.2011.09.033 22626509

[8] Choi HK Curhan G. Independent Impact of Gout on Mortality and Risk for Coronary Heart Disease. Circulation (2007) 116(8):894–900. doi: 10.1161/CIRCULATIONAHA.107.703389 17698728

[9] Yahyaoui R Esteva I Haro-Mora JJ Almaraz MC Morcillo S Rojo-Martinez G. Effect of Long-Term Administration of Cross-Sex Hormone Therapy on Serum and Urinary Uric Acid in Transsexual Persons. J Clin Endocrinol Metab (2008) 93(6):2230–3. doi: 10.1210/jc.2007-2467 18349066

[10] Stockl D Doring A Thorand B Heier M Belcredi P Meisinger C. Reproductive Factors and Serum Uric Acid Levels in Females From the General Population: The KORA F4 Study. PloS One (2012) 7(3):e32668. doi: 10.1371/journal.pone.0032668 22427861 PMC3302793

[11] Eun Y Kim IY Han K Lee KN Lee DY Shin DW. Association Between Female Reproductive Factors and Gout: A Nationwide Population-Based Cohort Study of 1 Million Postmenopausal Women. Arthritis Res Ther (2021) 23(1):304. doi: 10.1186/s13075-021-02701-w 34915918 PMC8675498

[12] Hak AE Choi HK. Menopause, Postmenopausal Hormone Use and Serum Uric Acid Levels in US Women–the Third National Health and Nutrition Examination Survey. Arthritis Res Ther (2008) 10(5):R116. doi: 10.1186/ar2519 18822120 PMC2592803

[13] Krishnan E Bennett M Chen L. Aging, Not Menopause, is Associated With Higher Prevalence of Hyperuricemia Among Older Women. Menopause (2014) 21(11):1211–6. doi: 10.1097/GME.0000000000000230 24714624

[14] Liao YY Chu C Wang Y Zheng WL Ma Q Hu JW. Long-Term Burden of Higher Body Mass Index From Childhood on Adult Cardiometabolic Biomarkers: A 30-Year Cohort Study. Nutr Metab Cardiovasc Dis (2021) 31(2):439–47. doi: 10.1016/j.numecd.2020.09.009 33223402

[15] Yokose C McCormick N Rai SK Lu N Curhan G Schwarzfuchs D. Effects of Low-Fat, Mediterranean, or Low-Carbohydrate Weight Loss Diets on Serum Urate and Cardiometabolic Risk Factors: A Secondary Analysis of the Dietary Intervention Randomized Controlled Trial (DIRECT). Diabetes Care (2020) 43(11):2812–20. doi: 10.2337/dc20-1002 PMC7576420 33082244

[16] Hwang J Lee MY Ahn JK Cha HS. Relationship Between Changing the Body Mass Index and Serum Uric Acid Alteration Among Clinically Apparently Healthy Korean Men. Arthritis Care Res (Hoboken) (2021). doi: 10.1002/acr.24576 33544980

[17] He H Pan L Ren X Wang D Du J Z Zhao J. Shan: The Effect of Body Adiposity and Alcohol Consumption on Serum Uric Acid: A Quantile Regression Analysis Based on the China National Health Survey. Front Nutr (2021) 8:724497. doi: 10.3389/fnut.2021.724497 35111792 PMC8801605

[18] He H Pan L Du J Jin Y Wang L Jia P. Effect of Fat Mass Index, Fat Free Mass Index and Body Mass Index on Childhood Blood Pressure: A Cross-Sectional Study in South China. Transl Pediatr (2021) 10(3):541–51. doi: 10.21037/tp-20-325 PMC8039780 33850812

[19] Freedman DS Wang J Maynard LM Thornton JC Mei Z Pierson RN. Relation of BMI to Fat and Fat-Free Mass Among Children and Adolescents. Int J Obes (Lond) (2005) 29(1):1–8. doi: 10.1038/sj.ijo.0802735 15278104

[20] Greendale GA Sternfeld B Huang M Han W Karvonen-Gutierrez C Ruppert K. Changes in Body Composition and Weight During the Menopause Transition. JCI Insight (2019) 4(5):e124865. doi: 10.1172/jci.insight.124865 PMC6483504 30843880

[21] Richiardi L Bellocco R Zugna D. Mediation Analysis in Epidemiology: Methods, Interpretation and Bias. Int J Epidemiol (2013) 42(5):1511–9. doi: 10.1093/ije/dyt127 24019424

[22] He H Pan L Pa L Cui Z Ren X Wang D. Shan: Data Resource Profile: The China National Health Survey (CNHS). Int J Epidemiol (2018) 47(6):1734–1735f. doi: 10.1093/ije/dyy151 30124853

[23] Johnson RJ Bakris GL Borghi C Chonchol MB Feldman D Lanaspa MA. Hyperuricemia, Acute and Chronic Kidney Disease, Hypertension, and Cardiovascular Disease: Report of a Scientific Workshop Organized by the National Kidney Foundation. Am J Kidney Dis (2018) 71(6):851–65. doi: 10.1053/j.ajkd.2017.12.009 PMC7286363 29496260

[24] Zhou BF. Predictive Values of Body Mass Index and Waist Circumference for Risk Factors of Certain Related Diseases in Chinese Adults–Study on Optimal Cut-Off Points of Body Mass Index and Waist Circumference in Chinese Adults. BioMed Environ Sci (2002) 15(1):83–96. 12046553

[25] Joo JK Hong GP Han SE Lee YJ Kim SC Kim CW. The Association Between Serum Uric Acid Level and Incidence of Metabolic Syndrome According to Menopausal Status in Korean Women. J Menopausal Med (2014) 20(3):126–32. doi: 10.6118/jmm.2014.20.3.126 PMC4286657 25580424

[26] Bubach S Horta BL Goncalves H Assuncao M. Early Age at Menarche and Metabolic Cardiovascular Risk Factors: Mediation by Body Composition in Adulthood. Sci Rep (2021) 11(1):148. doi: 10.1038/s41598-020-80496-7 33420216 PMC7794383

[27] Han T Meng X Shan R Zi T Li Y Ma H. Temporal Relationship Between Hyperuricemia and Obesity, and its Association With Future Risk of Type 2 Diabetes. Int J Obes (Lond) (2018) 42(7):1336–44. doi: 10.1038/s41366-018-0074-5 29717279

[28] Nielsen SM Bartels EM Henriksen M Wæhrens EE Gudbergsen H Bliddal H. Weight Loss for Overweight and Obese Individuals With Gout: A Systematic Review of Longitudinal Studies. Ann Rheum Dis (2017) 76(11):1870–82. doi: 10.1136/annrheumdis-2017-211472 PMC5705854 28866649

[29] Janssen I Powell LH Kazlauskaite R Dugan SA. Testosterone and Visceral Fat in Midlife Women: The Study of Women's Health Across the Nation (SWAN) Fat Patterning Study. Obes (Silver Spring) (2010) 18(3):604–10. doi: 10.1038/oby.2009.251 PMC2866448 19696765

[30] Lovejoy JC Champagne CM de Jonge L Xie H Smith SR. Increased Visceral Fat and Decreased Energy Expenditure During the Menopausal Transition. Int J Obes (Lond) (2008) 32(6):949–58. doi: 10.1038/ijo.2008.25 PMC2748330 18332882

[31] Guthrie JR Dennerstein L Taffe JR Lehert P Burger HG. The Menopausal Transition: A 9-Year Prospective Population-Based Study. The Melbourne Women's Midlife Health Project. Climacteric (2004) 7(4):375–89. doi: 10.1080/13697130400012163 15799609

[32] Marlatt KL Pitynski-Miller DR Gavin KM Moreau KL Melanson EL Santoro N. Body Composition and Cardiometabolic Health Across the Menopause Transition. Obes (Silver Spring) (2022) 30(1):14–27. doi: 10.1002/oby.23289 PMC8972960 34932890

[33] KJ R. Epidemiology: An Introduction. New York: Oxford University Press (2012).

[34] Jung JH Song GG Lee YH Kim JH Hyun MH Choi SJ. Serum Uric Acid Levels and Hormone Therapy Type: A Retrospective Cohort Study of Postmenopausal Women. Menopause (2018) 25(1):77–81. doi: 10.1097/GME.0000000000000953 28796699

[35] Cremonini E Bonaccorsi G Bergamini CM Castaldini C Ferrazzini S Capatti A. Metabolic Transitions at Menopause: In Post-Menopausal Women the Increase in Serum Uric Acid Correlates With Abdominal Adiposity as Assessed by DXA. Maturitas (2013) 75(1):62–6. doi: 10.1016/j.maturitas.2013.01.014 23415064

[36] Benjamin EJ Muntner P Alonso A Bittencourt MS Callaway CW Carson AP. Heart Disease and Stroke Statistics-2019 Update: A Report From the American Heart Association. Circulation (2019) 139(10):e56–528. doi: 10.1161/CIR.0000000000000659 30700139

