# Supplementary material for: The Mediation Effect of Body Composition on the Association Between Menopause and Hyperuricemia: Evidence From China National Health Survey
Source: Front Endocrinol (Lausanne). 2022 Jun 10;13:879384. doi: 10.3389/fendo.2022.879384 (PMC9226682; doi:10.3389/fendo.2022.879384)
Supplement: Supplementary file 1 [file DataSheet_1.docx]

Table S1 Basic characteristics in the age-matched data set (n=6202).

| Characteristics | HUA (n=2070) | | Non-HUA (n=4132) | | P |
| --- | --- | --- | --- | --- | --- |
| **Demographic information** |  |  |  |  |  |
| Age, year (median, IQR) | 54.4 | 17.4 | 54.4 | 17.3 | 0.688 |
| Marital status (n, %) |  |  |  |  | 0.664 |
| Unmarried | 85 | 4.11 | 164 | 3.97 |  |
| Inmarriage | 1763 | 85.17 | 3505 | 84.83 |  |
| Others | 220 | 10.63 | 457 | 11.06 |  |
| Residential areas (n, %) |  |  |  |  | <0.001 |
| Urban | 1489 | 71.93 | 2576 | 62.34 |  |
| Rural | 578 | 27.92 | 1548 | 37.46 |  |
| Education (n, %) |  |  |  |  | 0.003 |
| Illiterate/Elementary school | 714 | 34.49 | 1602 | 38.77 |  |
| High school | 942 | 45.51 | 1812 | 43.85 |  |
| College or above | 410 | 19.81 | 708 | 17.13 |  |
| Annual personal income (CHY) (n, %) |  |  |  |  | <0.001 |
| <10000 | 481 | 23.24 | 1109 | 26.84 |  |
| 10000- | 934 | 45.12 | 1865 | 45.14 |  |
| 30000- | 465 | 22.46 | 770 | 18.64 |  |
| ≥50000 | 147 | 7.10 | 240 | 5.81 |  |
| **Health related life-style factors** |  |  |  |  |  |
| Physical activity (n, %) |  |  |  |  | 0.436 |
| Low | 446 | 21.55 | 881 | 21.32 |  |
| Moderate | 1475 | 71.26 | 2899 | 70.16 |  |
| Heavy | 149 | 7.20 | 352 | 8.52 |  |
| Ever smoke (n, %) | 201 | 9.71 | 460 | 11.13 | 0.216 |
| Never smoke (n, %) | 1869 | 90.29 | 3666 | 88.72 |  |
| Ever-alcohol drink (n, %) | 493 | 23.82 | 980 | 23.72 | 0.002 |
| Never drink (n, %) | 1576 | 76.14 | 3145 | 76.11 |  |
| **Body composition indexes** |  |  |  |  |  |
| BMI (kg/m^2^) (median, IQR) | 25.63 | 4.78 | 23.39 | 4.50 | <0.001 |
| BMI category (n, %) |  |  |  |  |  |
| Under/normal weight | 670 | 32.37 | 2393 | 57.91 | <0.001 |
| Overweight | 868 | 41.93 | 1323 | 32.02 |  |
| Obesity | 532 | 25.70 | 416 | 10.07 |  |
| Body fat percentage (%) (median, IQR) | 36.3 | 7.0 | 32.6 | 7.8 | <0.001 |
| Fat free mass (kg) (median, IQR) | 37.5 | 4.6 | 36.1 | 4.6 | <0.001 |
| Fat mass (kg) (median, IQR) | 22.6 | 8.7 | 18.6 | 8.0 | <0.001 |
| FMI (kg/m^2^) (median, IQR) | 9.33 | 3.55 | 7.63 | 3.26 | <0.001 |
| FFMI (kg/m^2^) (median, IQR) | 15.33 | 1.15 | 14.89 | 1.23 | <0.001 |
| **Reproductive factors** |  |  |  |  |  |
| Menarche age |  |  |  |  |  |
| ≤12 | 222 | 10.72 | 353 | 8.54 | <0.001 |
| >12 | 1829 | 88.36 | 3728 | 90.22 |  |
| Post-menopause (n, %) | 782 | 37.78 | 1635 | 39.57 | 0.060 |
| Pre-menopause (n, %) | 1288 | 62.22 | 2497 | 60.43 |  |
| **Clinical characteristics** |  |  |  |  |  |
| eGFR (ml/min/1.73m^2^) (median, IQR) | 87.70 | 25.62 | 98.67 | 26.75 | <0.001 |
| Creatinine (μmol/L) (median, IQR) | 68.5 | 14.9 | 62.3 | 12.7 | <0.001 |

Table S2. Mediation analysis of body composition on the association between menopause and hyperuricemia.

| Mediator | Natural direct effect (NDE) | | | Natural indirect effect (NIE) | | | Total effect | | | % Mediated | | |
| --- | --- | --- | --- | --- | --- | --- | --- | --- | --- | --- | --- | --- |
|  | OR | 95%CI | | OR | 95%CI | | OR | 95%CI | | % | 95%CI | |
| BMI | 1.12 | 0.97 | 1.27 | 1.09 | 1.04 | 1.13 | 1.22 | 1.05 | 1.39 | 45.32 | 15.62 | 75.02 |
| Overweight/obesity | 1.11 | 0.97 | 1.25 | 1.07 | 1.04 | 1.11 | 1.19 | 1.04 | 1.35 | 43.16 | 12.30 | 74.02 |
| BFP | 1.05 | 0.90 | 1.21 | 1.23 | 1.16 | 1.29 | 1.29 | 1.10 | 1.49 | 81.70 | 40.65 | 100 |
| High BFP | 1.08 | 0.93 | 1.24 | 1.14 | 1.10 | 1.18 | 1.23 | 1.05 | 1.41 | 63.53 | 23.91 | 100 |
| FMI | 1.08 | 0.94 | 1.23 | 1.13 | 1.08 | 1.18 | 1.22 | 1.05 | 1.40 | 62.23 | 24.29 | 100 |
| High FMI | 1.08 | 0.94 | 1.22 | 1.10 | 1.07 | 1.13 | 1.18 | 1.03 | 1.34 | 58.00 | 16.37 | 99.63 |
| FFMI | **1.24** | **1.08** | **1.41** | **0.99** | **0.96** | **1.02** | 1.23 | 1.07 | 1.40 | **-5.44** | **-25.47** | **14.60** |
| High FFMI | **1.20** | **1.05** | **1.36** | **1.01** | **0.98** | **1.03** | 1.21 | 1.05 | 1.37 | **4.45** | **-10.10** | **19.00** |

Table S3. Sensitivity analysis on mediation analysis of body composition on the association between menopause and hyperuricemia, restricted in women experiencing menopausal transition within 5 years.

| Mediator | Natural direct effect (NDE) | | | Natural indirect effect (NIE) | | | Total effect | | | % Mediated | | |
| --- | --- | --- | --- | --- | --- | --- | --- | --- | --- | --- | --- | --- |
|  | OR | 95%CI | | OR | 95%CI | | OR | 95%CI | | % | 95%CI | |
| BMI | 1.16 | 0.95 | 1.38 | 1.13 | 1.06 | 1.21 | 1.32 | 1.07 | 1.57 | 48.34 | 17.50 | 79.18 |
| Overweight/obesity | 1.20 | 0.99 | 1.41 | 1.08 | 1.03 | 1.13 | 1.29 | 1.06 | 1.53 | 32.74 | 25.00 | 49.47 |
| BFP | 1.09 | 0.88 | 1.31 | 1.22 | 1.13 | 1.31 | 1.33 | 1.06 | 1.61 | 71.86 | 28.15 | 100 |
| High BFP | 1.10 | 0.89 | 1.32 | 1.17 | 1.12 | 1.23 | 1.29 | 1.04 | 1.55 | 64.58 | 20.73 | 100 |
| FMI | 1.15 | 0.94 | 1.36 | 1.16 | 1.08 | 1.23 | 1.33 | 1.07 | 1.59 | 54.72 | 21.64 | 87.80 |
| High FMI | 1.12 | 0.92 | 1.32 | 1.15 | 1.11 | 1.20 | 1.29 | 1.05 | 1.53 | 59.50 | 21.60 | 97.39 |
| FFMI | 1.26 | 1.03 | 1.49 | 1.06 | 1.00 | 1.11 | 1.33 | 1.08 | 1.59 | 22.26 | 1.49 | 43.02 |
| High FFMI | 1.20 | 1.05 | 1.36 | 1.01 | 0.98 | 1.03 | 1.21 | 1.05 | 1.37 | 4.45 | -10.10 | 19.00 |


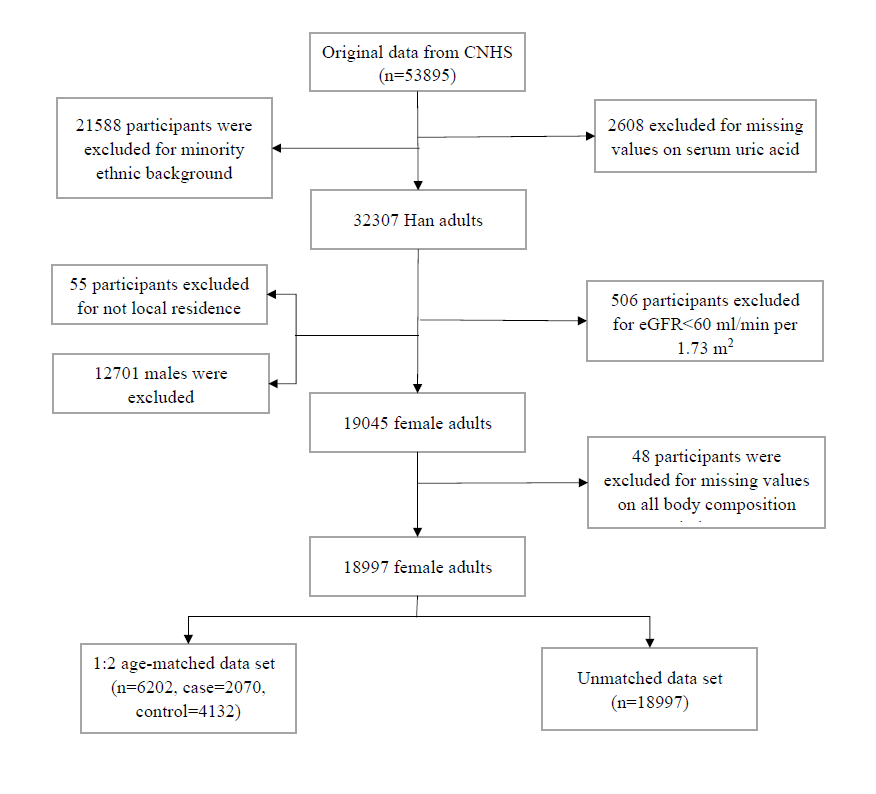


**Figure S1** The flow chart of the study.
